# Dynamics of brain valves: ostensible rectification mechanisms for cerebrospinal fluid flow

**DOI:** 10.1098/rsif.2025.0419

**Published:** 2025-10-29

**Authors:** Yisen Guo, Peter Aleksander Rousing Bork, Maiken Nedergaard, Douglas H. Kelley

**Affiliations:** ^1^ Department of Mechanical Engineering, University of Rochester, Rochester, NY, USA; ^2^ Center for Translational Neuromedicine, University of Copenhagen, Copenhagen, Denmark; ^3^ University of Rochester Center for Translational Neuromedicine, Rochester, NY, USA

**Keywords:** cerebrospinal fluid, perivascular spaces, perivascular pumping, glymphatic system

## Abstract

The flow of cerebrospinal fluid (CSF) through perivascular spaces (PVSs) is an important part of the brain’s system for clearing metabolic waste. Astrocyte endfeet ensheath the PVSs of penetrating arteries, separating them from brain extracellular space (ECS). Gaps between astrocyte endfeet could provide a low-resistance pathway for fluid transport across the endfoot wall. Recent research suggests that the astrocyte endfeet may also function as valves that rectify the CSF flow, allowing oscillatory pressures to drive net flows like those observed in experiments. In this study, we employ fluid–structure interaction modelling to investigate the endfoot valve mechanism. Due to the unavailability of precise *in vivo* measurements of gap shape and size, we explore three possible, though idealized, geometric arrangements: wedge-shaped gaps, overlapping endfeet of different sizes and curvature of the endfoot wall. For each, we quantify the dependence of net flow on oscillatory pressure amplitude, frequency and other key parameters. For all three, our simulations demonstrate effective flow rectification at frequencies associated with functional hyperaemia, respiration and cardiac pulsation.

## 1. Introduction

The clearance of metabolic waste from the brain, which is essential for maintaining neural health and cognitive function, is facilitated by the glymphatic system [1–4], a pathway enabling solute exchange between cerebrospinal fluid (CSF) and interstitial fluid (ISF) [5–7]. Fluid flow is key to the system, and substantial flows pass through the CSF-filled perivascular spaces (PVSs) that surround pial and penetrating blood vessels in the brain. In pial PVSs of anaesthetized mice, where flows have been observed directly with two-photon microscopy, the time-averaged CSF motion is parallel (not anti-parallel) to the blood, with a mean speed around 20 µm s^−1^. The speed fluctuates in synchrony with the cardiac pulsation of the adjacent artery wall, with fluctuations typically approximately half as fast as the mean [8,9]. Additionally, functional hyperaemia causes arteries to dilate and constrict as blood is rerouted to different brain regions. These vascular motions have amplitude nearly 10 times greater than those driven by cardiac pulsation, but at much lower frequency, and similarly cause corresponding CSF motions [10], as predicted by models [11]. Artery wall motion seems to be a major driver of flow in PVSs.

 However, the mechanism by which artery wall motion drives directional flow parallel to the blood, as opposed to purely fluctuating flow, remains uncertain. Intuition and mass conservation dictate that CSF must flow away from narrowing regions of PVS and towards widening regions of PVS, but they dictate no preference for displacement parallel or anti-parallel to the direction of blood flow. If peristalsis were responsible for pumping along PVSs, fluid would be displaced preferentially in the direction of propagation of artery wall deformation [8,12–14]. However, because cardiac pulsations propagate orders of magnitude faster than CSF, theory predicts negligible pumping by peristalsis [15], and simulations agree [16,17]. Another suggested mechanism is impedance pumping [10], in which directional flow arises from local deformations of a vessel boundary because the resulting propagating waves reflect and interfere [18–20]. However, the directional flow driven by impedance pumping is so sensitive to the pulsation frequency and the locations of reflection sites that it may not be robust in a complex biological system. A canonical idea is that CSF is produced by choroid plexus tissue lining the brain ventricles and taken up by arachnoid villi in the subarachnoid space surrounding the brain, implying a directional flow. However, these functions of the choroid plexus and arachnoid villi have recently been questioned [7,17,21]. Additionally, if flow were driven solely by production of CSF at choroid plexus and uptake at arachnoid villi, we would expect CSF to pass directly through the low-resistance subarachnoid space without entering the higher-resistance perivascular spaces.

In other biological circulation systems, the flow direction is set by mechanical valves, like those present in hearts, veins and lymph vessels, which rectify fluctuations to produce a mean flow. Though no mechanical valves have been visually detected in the glymphatic system, researchers have wondered if valve action might occur anyway, especially at gaps between astrocyte endfeet [3,4,22]. Penetrating PVSs are ensheathed by astrocyte endfeet, which separate CSF-filled PVSs from surrounding, ISF-filled extracellular spaces (ECSs). Between the endfeet are gaps, which contribute to the selective permeability of the perivascular interface [23] and would allow exchange of CSF and ISF, perhaps involving bulk flow. Because the gaps are narrow, small changes in their widths could induce major changes in glymphatic flow; bottlenecks are good places for flow control. Additionally, astrocyte endfeet have cell membranes that are densely decorated with aquaporin-4 (AQP4) water channels. Genetically knocking out AQP4 reduces glymphatic function [24], especially for large molecules [25], as does distributing AQP4 uniformly across astrocytes, without preference for endfeet [26]. Though the exact mechanism is uncertain, the fact that AQP4 facilitates glymphatic function and is prevalent on endfeet suggests that endfeet might control flow.

Unfortunately, endfoot gaps are too small to see in detail with optical microscopes. Electron microscopes resolve gaps effectively but require sample fixation methods that deform the gaps substantially and somewhat unpredictably. *In vivo* imaging has measured endfoot pulsations in mouse cortex of the order of 1 µm during sleep, though gaps were not resolved [27]. Lacking *in vivo* observations of gap shapes or dynamics, researchers have instead used computational models to explore the possibility that gaps may act as valves. If gaps are wedge-shaped, they would widen and therefore facilitate exchange when the endfeet bend in one direction but narrow and hinder exchange when the endfeet bend in the opposite direction, thus favouring one flow direction over the other [3]. Separately, curvature of the endfoot wall implies that expansion of gaps due to high pressure in the PVS is greater than contraction of gaps due to low pressure in the PVS, favouring flow from PVS to ECS [22]. Regardless of the precise mechanism, if gaps do act as valves, simulations suggest they could produce mean flows and pulsations of the same order of magnitude as those observed *in vivo* [4]. The idea that endfeet might help control glymphatic flows is further supported by experimental results indicating that astrocytes are sensitive to shear stresses below 0.1 dyn cm^−^${,}^{2}$ [28].

The presence of valves in the glymphatic system might serve not only to ensure directional flow but also to protect brain tissue from violently fast flows. The brain is more complex than any other organ or machine known to exist—and is correspondingly delicate. Neural signals propagate across synapses via the motion of molecular neurotransmitters. Because synapses are part of the ECS, fast flow there could sweep away neurotransmitters, interfering with signalling. The sensitivity of synapses to flow may explain why the glymphatic system functions primarily during sleep [29]. Additionally, one experimental study found that when pressure waves propagated across the brain, their amplitude correlated with endfoot damage [30]. Valves that narrow or close to prevent violent flow could preserve the function of deeper brain tissue.

In this study, we build on prior, foundational models by using two- and three-dimensional fluid–structure interaction simulations to examine three geometric arrangements that could facilitate flow rectification: wedge-shaped gaps, overlapping endfeet of unequal lengths and curved endfeet. Rectification by overlapping endfeet of unequal lengths has not been suggested before but has precedent elsewhere in the body: on lymphatic vessels, endothelial cells have overlaps that are dynamically regulated in response to fluid volume changes [31]. The geometric arrangements we consider are highly idealized, with shapes and structures much simpler than those of actual cells, to facilitate exploration of basic mechanisms. We ask how well each of the arrangements can rectify flow. We then analyse how rectification depends on frequency, pressure amplitude, gap angle and spatial arrangement, aiming to identify robust configurations that could feasibly function as valves. We determine the critical pressures at which the valves would close, potentially protecting the ECS from violent flow. Our work contributes to a mechanistic understanding of how brain microanatomy may support the unidirectional movement of CSF, thereby promoting effective glymphatic clearance and protecting neural tissue from waste accumulation.

## 2. Numerical methods

We used the finite element code COMSOL Multiphysics to model the flow of CSF through the gaps between astrocyte endfeet. CSF was taken to be an incompressible, viscous fluid with the properties of water at 37${,}^{\circ }$C: density $10^{3}$ kg m^−^${,}^{3}$ and viscosity $0.7\times 10^{-3}$ Pa s. The incompressible Navier–Stokes equations were solved to determine the flow field. To drive flow, a pressure difference was applied between the inflow and outflow surfaces. The remaining exterior boundary conditions were assumed to be symmetry. Where CSF contacted endfeet, a no-slip condition was applied.

The endfeet were modelled as isotropic, linear elastic solids. The geometry and material properties used in all simulations were the same as in Bork *et al.* [3]. In all simulations, except the code validation case, the following values were used: thickness 2 µm, minimum initial gap 0.25 µm, Poisson’s ratio 0.5 and Young’s modulus 3.2 kPa. The endfeet were cantilevered at the outer walls of the computational domain and allowed to deform under the pressure of fluid.

The fluid and the solid domains were fully coupled at their boundaries, i.e. the equations were solved simultaneously at each time step. An arbitrary Lagrangian–Eulerian formulation was used for accommodating geometric changes of the fluid domain. The fluid equations were solved on a Eulerian domain with a moving mesh, and to avoid collapse of the mesh between endfeet, a small separation between endfeet was used to represent a fully closed valve. Therefore, there was some small flow rate even when the valves were closed, but this flow rate was negligible.

We first validated our numerical results by comparing with the two-dimensional analytical solutions in Bork *et al.* [3], and good agreement was achieved (figure 6). Mesh sensitivity studies were also performed to ensure that the meshes were sufficiently fine to resolve the computational domains and that the numerical results did not change substantially when the mesh size was decreased further (figure 7). Detailed results of code validation and mesh sensitivity studies are presented in appendix A.

## 3. Results

To investigate the hypothesized valve-like function of astrocyte endfeet, we simulated three geometric arrangements: a wedge-shaped gap, endfeet of unequal length and endfeet with curvature.

### 3.1. Simulations validate prior analysis: wedge-shaped gaps can rectify flow

**Figure 1.**
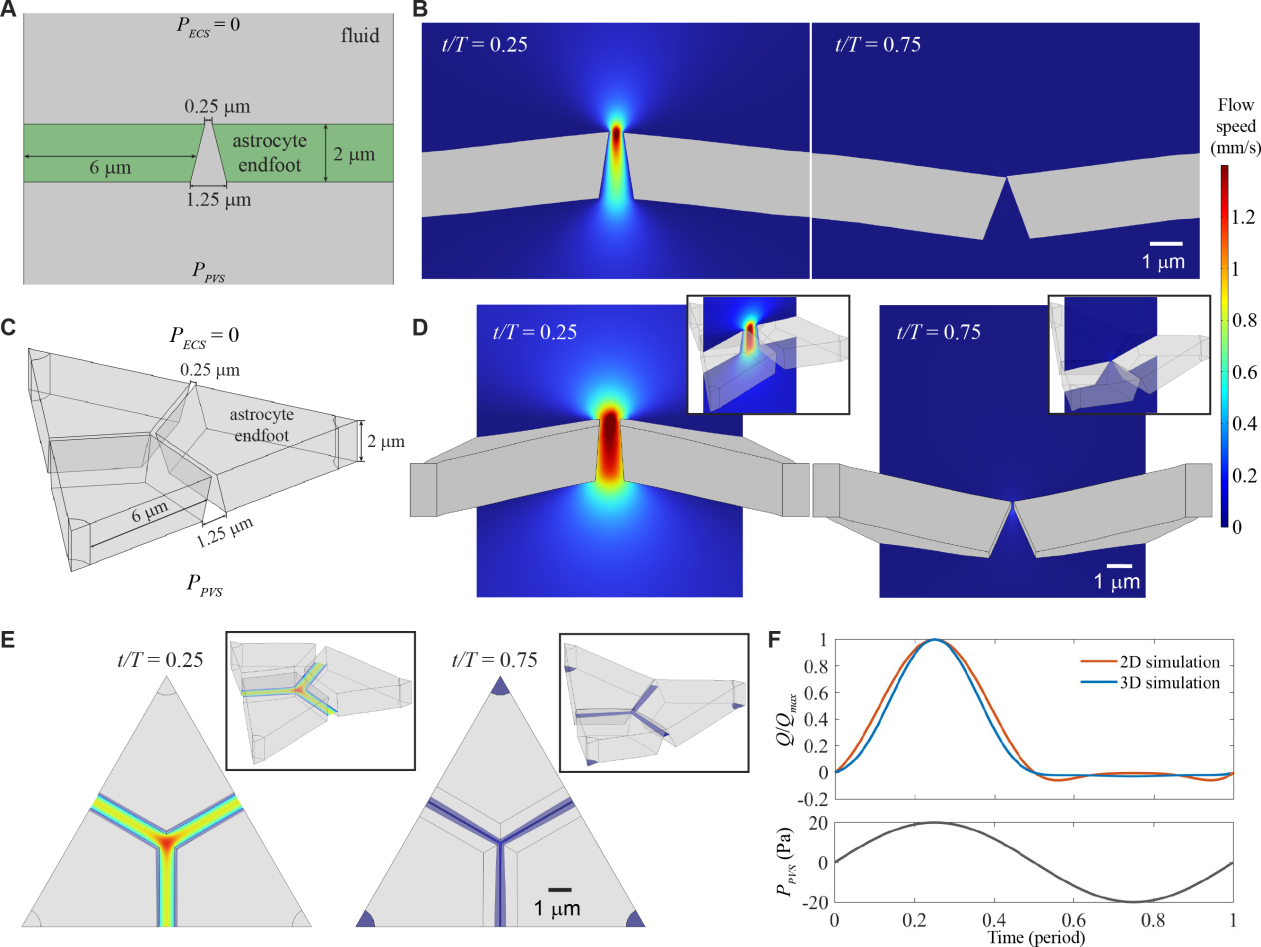
Wedge-shaped gaps between elastic endfeet can rectify flow in two- and three-dimensional numerical simulations. (A) In two-dimensional simulations of a wedge-shaped gap between two endfeet, we modelled the pressure in the extracellular space (ECS) as $P_{\mathrm{ECS}}=0$ and the pressure in the perivascular space as $P_{\mathrm{PVS}}$, which oscillated sinusoidally with period T and pressure amplitude $p_{\mathrm{amplitude}}=20$ Pa (see panel F). (B) The endfeet bent under pressure, widening the gap and allowing fast flow when fluid was passing into the ECS, but closing the gap and blocking flow when fluid was passing in the opposite direction. (C) We set up three-dimensional simulations similar to those in two dimensions and further explored a corner where three endfeet abutted. (D) As in two dimensions, the endfeet bent under pressure in a way that rectified flow. (E) When $P_{\mathrm{PVS}}$ was maximum, flow was fastest at the corner; when $P_{\mathrm{PVS}}$ was most negative, flow was slowest there. (F) The normalized volume flow rate through the gap $Q/Q_{\max}$ varied over time, showing efficient rectification and good agreement with the theoretical predictions of [3] (see figure 6). Figures show only the regions near the endfeet, full computational domains extend further to ensure parallel streamlines at the inlets and outlets.

To test the valve mechanism reported previously [3], we simulated flow through two- and three-dimensional wedge-shaped gaps, driven by a pressure difference $P_{\mathrm{PVS}}-P_{\mathrm{ECS}}$ that oscillated sinusoidally between −20 and 20 Pa with period T, as shown in figure 1A,C. The two-dimensional simulations showed that endfeet deformed and CSF flowed in response to the applied pressure. The gap widened, and fast flow passed through it, when $P_{\mathrm{PVS}}$ was maximum (at time t/T=0.25). When $P_{\mathrm{PVS}}$ was most negative (at time t/T=0.75), the gap closed and flow was blocked; see figure 1B. More specifically, the gap closed whenever $P_{\mathrm{PVS}}\leq -18.6$ Pa. Throughout the oscillation period, flow from PVS to ECS was faster than flow in the opposite direction, as shown in electronic supplementary material, video S1. A wedge-shaped gap between flexible endfeet tended to rectify oscillatory flow, as seen in prior analysis [3].

In three dimensions, three or more endfeet can abut at a point, forming a corner, though no such corners are possible in two dimensions. In our three-dimensional simulations, therefore, we explored the effects of a corner, as shown in figure 1. As in two dimensions, fast flow passed through a wide gap when $P_{\mathrm{PVS}}$ was maximum (at time t/T=0.25), but flow was much slower, and the gap was much narrower, when $P_{\mathrm{PVS}}$ was most negative (at time t/T=0.75; see figure 1D). Though the three-dimensional gap never entirely closed, the endfeet were in contact whenever $P_{\mathrm{PVS}}\leq -5.5$ Pa. Throughout the oscillation period, flow from PVS to ECS was faster than flow in the opposite direction, as shown in electronic supplementary material, video S2. Near the corner, when $P_{\mathrm{PVS}}$ was maximum, the gap widened more and allowed faster flow than elsewhere, but when $P_{\mathrm{PVS}}$ was most negative, the gap narrowed more and kept flow slower than elsewhere, as shown in figure 1E. Thus, rectification was stronger near the corner. Flow can be quantified with Q, the instantaneous volume flow rate from PVS to ECS, whose variation over the oscillation period is shown in figure 1F, normalized by its maximum value, $Q_{\max}$. For two- and three-dimensional simulations, Q increased with $P_{\mathrm{PVS}}$ when $P_{\mathrm{PVS}}>0$, but Q dropped to near zero when $P_{\mathrm{PVS}}<0$, demonstrating rectification.

Our results broadly agree with the analytic predictions of Bork *et al.* [3]. Simulations using dimensions exactly matching the ‘large’ and ‘small’ cases of Bork *et al.* agreed quite closely with the theoretical predictions of those authors, except when $P_{\mathrm{PVS}}$ was most negative, as shown in figure 6 in appendix A. The mismatch may stem from the fact that the theoretical predictions neglected stretching and the effects of shear forces on the endfeet, which leads to early close of the gap when $P_{\mathrm{PVS}}<0$.

### 3.2. Endfeet of unequal length can rectify flow

**Figure 2.**
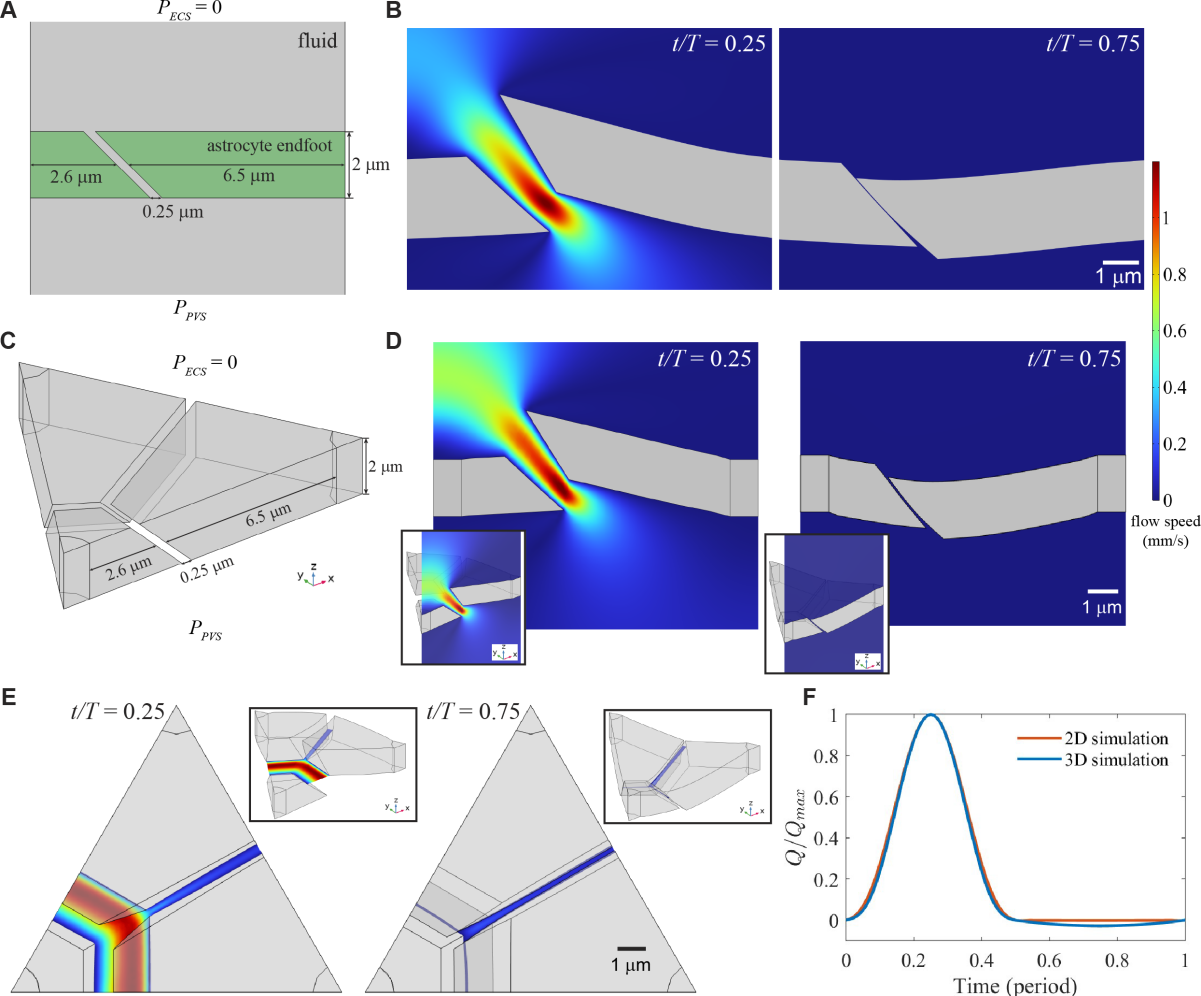
Pairs of long and short elastic endfeet can rectify flow in two- and three-dimensional numerical simulations. (A) In two-dimensional simulations, we considered an angled gap between endfeet of different lengths, with pressure $P_{\mathrm{ECS}}=0$ in the extracellular space (ECS) and pressure $P_{\mathrm{PVS}}$ in the perivascular space (PVS) varying sinusoidally. (B) When $P_{\mathrm{PVS}}>P_{\mathrm{ECS}}$, the endfeet bent to widen the gap, allowing fast flow into the ECS, but when $P_{\mathrm{PVS}}<P_{\mathrm{ECS}}$, the endfeet bent to close the gap, blocking flow. (C) In three-dimensional simulations, we again considered angled gaps between endfeet of different lengths, and further explored a corner where three endfeet abutted. (D) As in two dimensions, the endfeet bent under pressure in a way that rectified flow. (E) When $P_{\mathrm{PVS}}$ was maximum, flow was fastest near the corner; when $P_{\mathrm{PVS}}$ was most negative, flow was slowest there. (F) The normalized volume flow rate through the gap $Q/Q_{\max}$ varied over time, showing efficient rectification.

Endfoot sizes vary widely [32], so we speculate that flow could be rectified where a long endfoot meets a short endfoot if the two endfeet overlap, as sketched in figure 2A. In two-dimensional simulations, pressure differences between the PVS and ECS displaced the long endfoot more than the short endfoot (though both were equally thick and equally stiff, in the sense of having the same modulus). The gap was wide and flow there was fast when $P_{\mathrm{PVS}}$ was maximum (at time t/T=0.25), but the gap was narrower when $P_{\mathrm{PVS}}$ was negative and the gap closed when $P_{\mathrm{PVS}}\leq -2.4$ Pa (see figure 2B). As with a wedge-shaped gap, throughout the oscillation period, the gap expanded and contracted in a way that allowed more flow from the PVS to the ECS than in the opposite direction, as shown in electronic supplementary material, video S3.

In three dimensions, as with wedge-shaped gaps, corners can arise, and the endfeet abutting at a corner may overlap in several different configurations, in addition to having varying combinations of lengths. We studied the simple case of two long endfeet separated by a symmetric gap, both overlapping a small endfoot, as shown in figure 4C. Simulations produced results similar to those in two dimensions, as shown in figure 2D: when $P_{\mathrm{PVS}}$ was maximum (at time t/T=0.25), the gap was wide and flow was fast, but when $P_{\mathrm{PVS}}$ was most negative (at time t/T=0.75), the gap was narrow and flow was slow. In regions with overlapping endfeet, the gaps closed when $P_{\mathrm{PVS}}\leq -2.4$ Pa (see figure 2D). More details of the variation over time are shown in electronic supplementary material, video S4. Little flow passed through the symmetric gap between long endfeet, as shown in figure 2E, because deformation narrowed the gap and reduced flow. In both two and three dimensions, the normalized volume flow rate $Q/Q_{\max}$ increased with $P_{\mathrm{PVS}}$ when $P_{\mathrm{PVS}}>0$ but dropped near zero when $P_{\mathrm{PVS}}<0$, as shown in figure 2F.

### 3.3. Endfoot curvature can rectify flow

**Figure 3.**
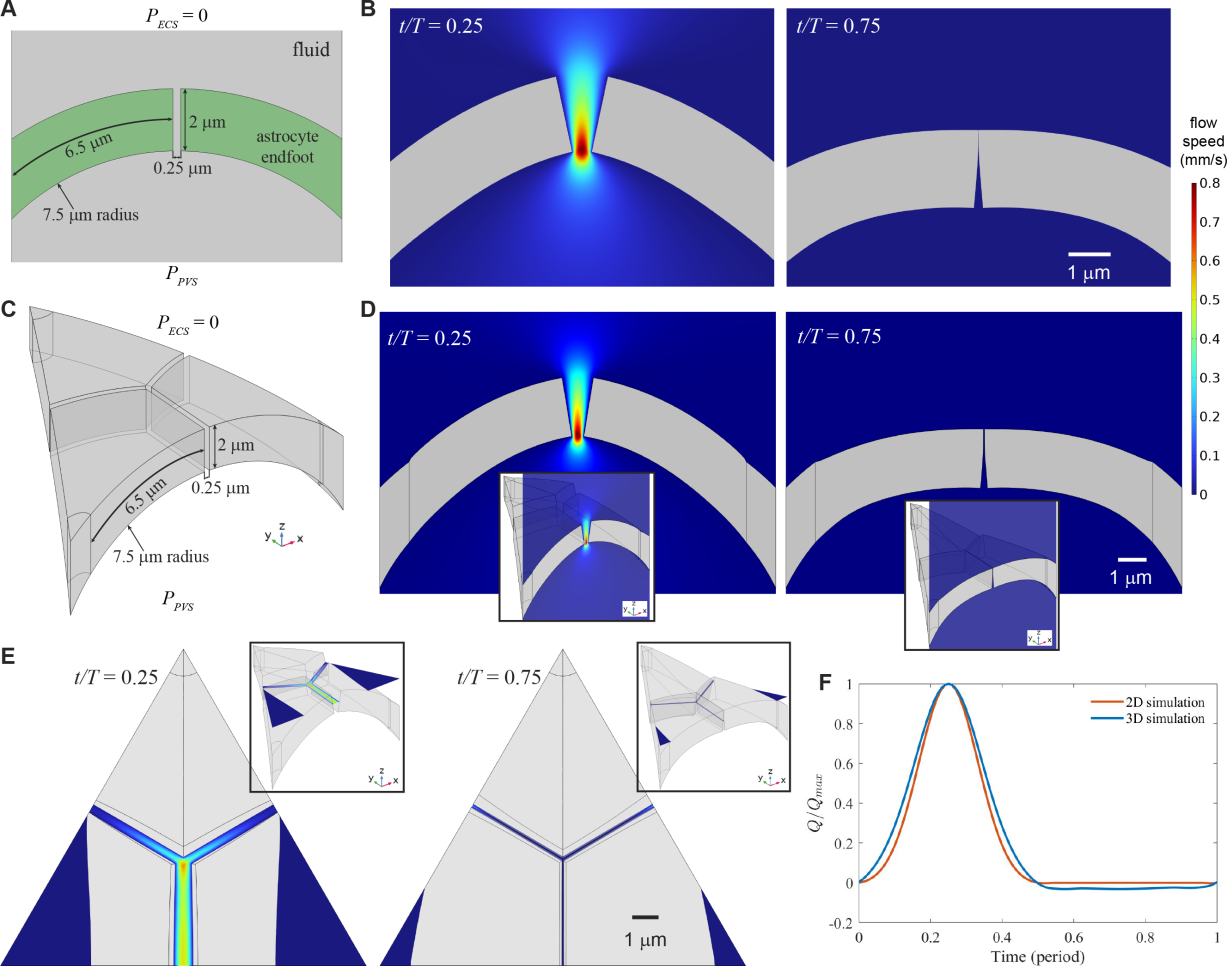
Curvature of perivascular spaces can rectify flow in two- and three-dimensional numerical simulations. (A) In two-dimensional simulations, we considered a uniform gap between two endfeet with circular curvature, with pressure $P_{\mathrm{ECS}}=0$ in the extracellular space (ECS) and $P_{\mathrm{PVS}}$ in the perivascular space (PVS) varying sinusoidally. (B) When $P_{\mathrm{PVS}}>P_{\mathrm{ECS}}$, the endfeet bent to widen the gap, allowing fast flow into the ECS, but when $P_{\mathrm{PVS}}<P_{\mathrm{ECS}}$, the endfeet bent to close the gap, blocking flow. (C) In three-dimensional simulations, we again considered gaps between curved endfeet, and further explored a corner where three endfeet abutted. (D) As in two dimensions, the endfeet bent under pressure in a way that rectified flow. (E) When $P_{\mathrm{PVS}}$ was maximum, flow was fastest near the corner and fast throughout the gap oriented parallel to the curvature axis; when $P_{\mathrm{PVS}}$ was most negative, flow was slowest near the corner and slow throughout the gap oriented parallel to the curvature axis. (F) The normalized volume flow rate through the gap $Q/Q_{\max}$ varied over time and showed efficient rectification.

Since penetrating PVSs have radii of the order of just 20 µm or less, their maximum principal curvature is correspondingly large, of the order of $5\times 10^{4}$ m${,}^{-1}$. Accordingly, a recent analytical study [22] raised the possibility that curvature and elastic stretching could combine to rectify flow through gaps in the PVS wall, and we have performed simulations to corroborate their analytical model. Bending, however, was not considered in that study. We used simulations to explore the combined effects of curvature, stretching and bending in response to oscillating pressure, starting with the two-dimensional configuration sketched in figure 3A. Similar to the cases of wedge-shaped gaps and overlaps between long and short endfeet, two-dimensional simulations of curved endfeet showed fast flow through an enlarged gap when $P_{\mathrm{PVS}}$ was maximum (at time t/T=0.25) and a closed gap that blocked flow when $P_{\mathrm{PVS}}$ was most negative, and the gap closed when $P_{\mathrm{PVS}}\leq -6.1$ Pa (see figure 3B). More details are shown in electronic supplementary material, video S5.

In three dimensions, the orientation of gaps on the endfoot wall might come into play. For example, gaps parallel to the curvature axis (or equivalently, the axis of the cylindrical perivascular space, in this idealized model), grow when the PVS expands radially, but gaps perpendicular to the curvature axis grow when the PVS is extended axially. We explored a three-dimensional arrangement of three endfeet with one gap parallel to the curvature axis (equivalent to the gap in the two-dimensional case) and two gaps at oblique angles to the axis, as shown in figure 3C. First, consider the gap parallel to the curvature axis. Simulations showed that it expanded and allowed fast flow when $P_{\mathrm{PVS}}$ was maximum (at time t/T=0.25), but closed when $P_{\mathrm{PVS}}$ was most negative (at time t/T=0.75), and the gap closed when $P_{\mathrm{PVS}}\leq -6.1$ Pa (see figure 3D). Simulations also showed more efficient rectification there than in the oblique gaps—faster flow when $P_{\mathrm{PVS}}$ was maximum and slower flow when $P_{\mathrm{PVS}}$ was most negative—as shown in figure 3E and electronic supplementary material, video S6.

The normalized volume flow rate $Q/Q_{\max}$, plotted in figure 3F, increased with $P_{\mathrm{PVS}}$ when $P_{\mathrm{PVS}}>0$, for both the two- and three-dimensional simulations. When $P_{\mathrm{PVS}}<0$, $Q/Q_{\max}∼0$ in two-dimensional simulations, but in three-dimensional simulations, flow persisted at nearly 20% of its maximum value. That phenomenon is consistent with the fact that the oblique gaps opened and closed less than the gap parallel to the curvature axis. When $P_{\mathrm{PVS}}$ was most negative, the flow speed in the oblique gaps reached 60% of its value when $P_{\mathrm{PVS}}$ was maximum. Whether curvature can provide an effective valve-mechanism therefore depends on the orientation of gaps between endfeet.

### 3.4. Comparing the three geometric arrangements

Having found that wedge-shaped gaps, unequal endfoot lengths and wall curvature can all rectify flow from PVS to ECS, we set out to compare the effectiveness of rectification in the three arrangements. To do so, we performed three-dimensional simulations using the geometries in figures 1–3, keeping the endfoot thickness at 2 µm, the initial smallest gap at 0.25 µm, and the endfoot length at 6 µm (with the longer endfoot set to 6 µm in the unequal-length case) across all three geometric arrangements. First, we wondered how their effectiveness might depend on the frequency of oscillation. Pressure oscillations with periods ranging from minutes to fractions of a second have been proposed to drive fluid flow, and stimulation with functional ultrasound (involving periods of tens of microseconds) has been reported to promote flow [33–36]. Could the same valve mechanism rectify flow across all these frequencies?

**Figure 4.**
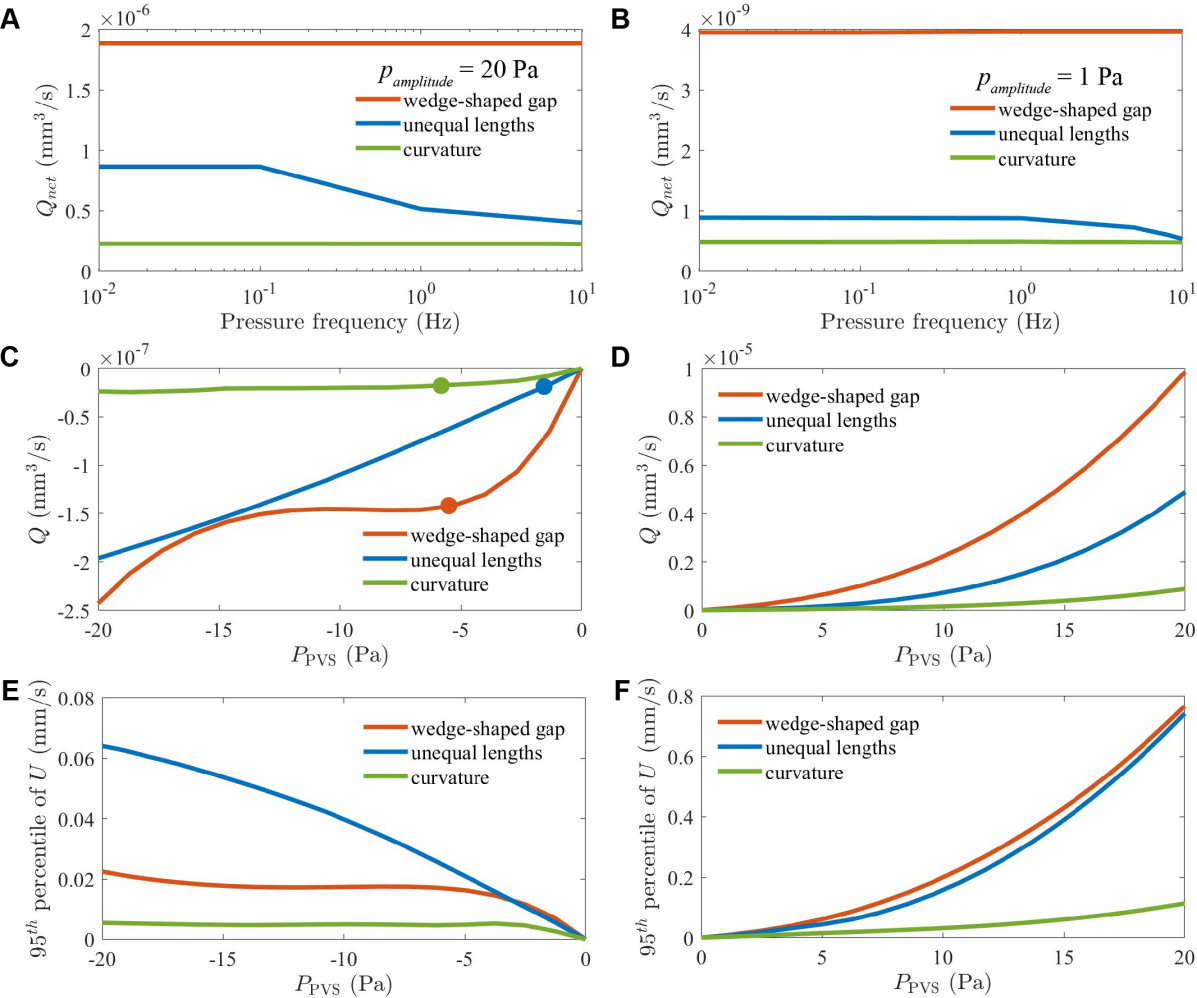
Effects of varying frequency and pressure amplitude. (A,B) When the pressure $P_{\mathrm{PVS}}$ oscillated, the net volume flow rate $Q_{\mathrm{net}}$ depended only weakly on the oscillation frequency, regardless of whether the pressure amplitude $p_{\mathrm{amplitude}}$ was 20 or 1 Pa. $Q_{\mathrm{net}}$ decreased slightly at high frequencies for endfeet of unequal length. (C–F) Volume flow rate Q and 95th percentile of velocity in steady three-dimensional simulations. When $P_{\mathrm{PVS}}<0$, $Q_{\mathrm{net}}$ had smallest magnitude (was least negative) through curved PVS walls and had largest magnitude through a wedge-shaped gap (C). Dots indicate pressures at which endfeet made contact. Accordingly, the velocity magnitude was smallest through curved PVS walls and largest through a wedge-shaped gap (E). When $P_{\mathrm{PVS}}>0$, $Q_{\mathrm{net}}$ had much larger magnitude generally, and most of all for flow through a wedge-shaped gap. Accordingly, the velocity was fastest through a wedge-shaped gap (F).

As shown in figure 4A, in simulations with a 20 Pa oscillation amplitude, a wedge-shaped gap produced a time-averaged net flow rate $Q_{\mathrm{net}}$ approximately twice as large as that produced by endfeet of unequal lengths, and approximately four times as large as that produced by wall curvature. For frequencies ranging from 0.01 Hz to 10 Hz, $Q_{\mathrm{net}}$ was essentially constant for a wedge-shaped gap and for wall curvature. For endfeet of unequal length, $Q_{\mathrm{net}}$ dropped slightly at high frequencies. In similar simulations with a 1 Pa oscillation amplitude, $Q_{\mathrm{net}}$ changed even less, for all three arrangements, as shown in figure 4B.

We also wondered how the effectiveness of rectification might depend on the pressure in the PVS. Steady simulations with $P_{\mathrm{PVS}}<0$ showed that fluid flowed from ECS into PVS and that the volume flow rate Q had greatest amplitude (was most negative) with a wedge-shaped gap and smallest amplitude (least negative) with wall curvature (figure 4C). With a wedge-shaped gap, Q varied little over the range $-5\mathrm{Pa}\geq P_{\mathrm{PVS}}\geq -14\mathrm{Pa}$, when the endfeet were in contact. With unequal endfoot lengths or wall curvature, endfoot contact altered the variation of Q with $P_{\mathrm{PVS}}$ less dramatically. Steady simulations with $P_{\mathrm{PVS}}>0$ showed that fluid flowed from PVS into ECS and that the flow rate Q was typically larger by almost two orders of magnitude than in simulations with $P_{\mathrm{PVS}}<0$ (figure 4D). Again, Q had greatest amplitude with a wedge-shaped gap and smallest amplitude with wall curvature. For all three arrangements, Q increased with $P_{\mathrm{PVS}}$ monotonically and faster than linear.

In the same simulations, we quantified flow using $U_{95}$, the 95th percentile of the flow speed U. For all three arrangements, $U_{95}$ increased as the magnitude of $P_{\mathrm{PVS}}$ increased (figure 4E). Interestingly, over the range $0\geq P_{\mathrm{PVS}}\geq -3.1$ Pa, $U_{95}$ was greater with a wedge-shaped gap than with endfeet of unequal length, but over the range $P_{\mathrm{PVS}}<-3.1$ Pa, the opposite was true. Thus, for $P_{\mathrm{PVS}}<-3.1$ Pa, the highest-speed regions were faster with endfeet of unequal length than with a wedge-shaped gap, though the mean speed (which is proportional to flow rate Q) was lower with endfeet of unequal length than with a wedge-shaped gap. $U_{95}$ was much smaller with a curved PVS wall than with a wedge-shaped gap or with endfeet of unequal length. When $P_{\mathrm{PVS}}>0$, $U_{95}$ was typically larger by an order of magnitude than when $P_{\mathrm{PVS}}<0$ (figure 4F). $U_{95}$ was greatest with a wedge-shaped gap and least with a curved wall. For all three arrangements, $U_{95}$ increased with $P_{\mathrm{PVS}}$ monotonically and faster than linear.

### 3.5. Modelling a cylindrical wall of endfeet

**Figure 5.**
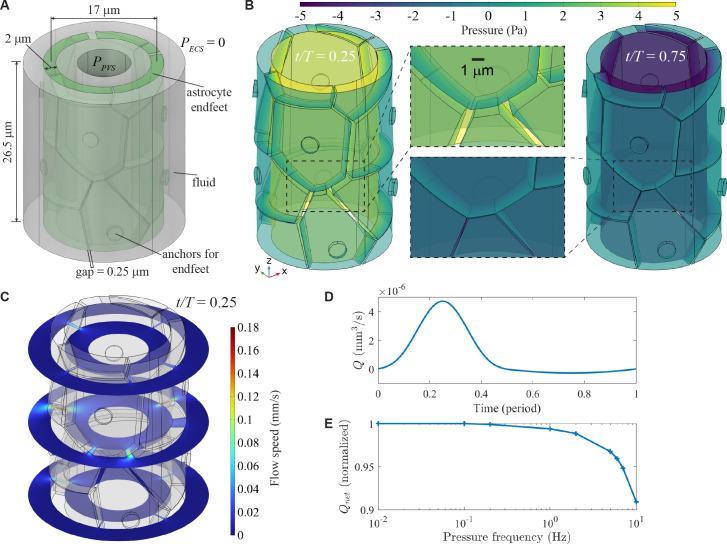
Model of a cylindrical wall of endfeet. (A) Schematic of the simulation set-up. $P_{\mathrm{PVS}}$ changes sinusoidally with an amplitude of 5 Pa. (B) Endfeet deformations at t/T=0.25 and t/T=0.75, corresponding to the maximum and most negative values of $P_{\mathrm{PVS}}$, respectively. (C) Flow speed distribution at three slices at t/T=0.25. (D) Flow rate (Q) over one period of the sinusoidal pressure waveform (frequency = 0.01 Hz). The net flow rate $Q_{\mathrm{net}}$ is $0.969\times 10^{-6}$ mm${,}^{3}$ s^−1^. (E) $Q_{\mathrm{net}}$ decreases slightly with increasing pressure frequency (0.01−10 Hz).

**Figure 6.**
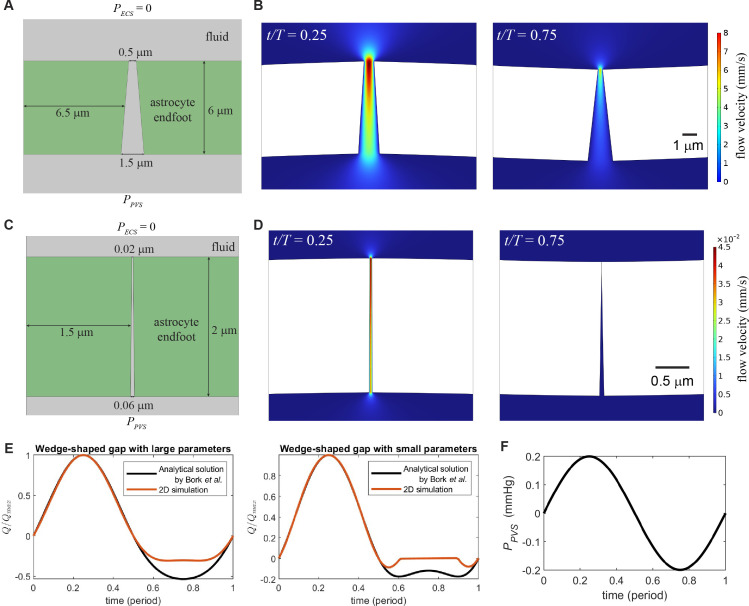
Simulated rectification by two-dimensional wedge-shaped gaps closely matches theory. Two-dimensional simulations using the dimensions shown in (A), which exactly match the ‘large’ case in [3], and applying the pressure variation shown in (F), predict a wide gap and fast flow when $P_{\mathrm{PVS}}$ is maximum, but a narrow gap and slow flow when $P_{\mathrm{PVS}}$ is most negative (B). The normalized volume flow rate $Q/Q_{\max}$ closely tracks the theoretical prediction of [3], except when $P_{\mathrm{PVS}}$ is strongly negative (E). Similarly, two-dimensional simulations using the dimensions shown in (C), which exactly match the ‘small’ case in [3], predict variations in gap size and speed (D), along with a normalized volume flow rate that closely tracks the theoretical prediction except when $P_{\mathrm{PVS}}$ is strongly negative (E).

**Figure 7.**
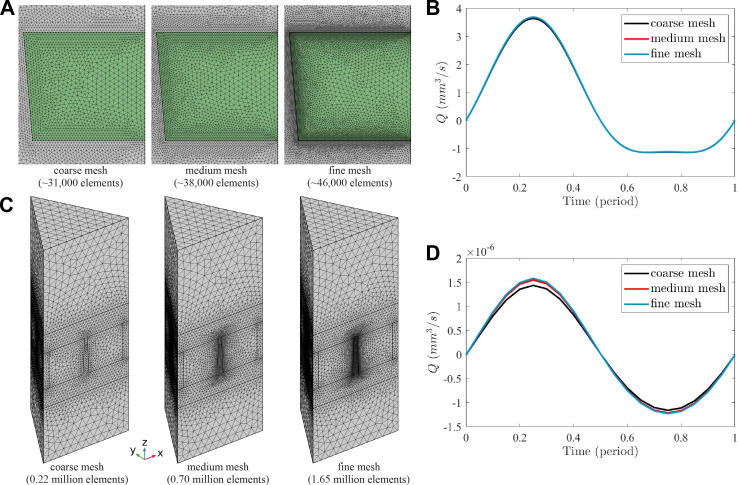
Mesh sensitivity studies. (A,B) Meshes of two-dimensional simulations based on the geometry in figure 6A. Only the regions near the endfeet are shown, the full computational domains extend much further to ensure parallel streamlines at the inlets and outlets. The element sizes of endfoot walls are 0.15, 0.1 and 0.05 µm for coarse, medium and fine meshes, respectively, with a maximum element size of 0.3 µm in the domain. Flow rates predicted with medium and fine meshes show negligible differences. (C,D) Meshes of three-dimensional simulations using the same geometry. The endfoot wall element sizes of the gaps are 0.3, 0.15 and 0.1 µm for coarse, medium, and fine meshes, respectively, with maximum element sizes of 0.5 µm near the endfeet and 1.2 µm far away from them. Again, flow rates predicted with medium and fine meshes show negligible differences.

Corner effects may be small and flows between pairs or triplets of endfeet may be dominated by macroscopic effects along whole perivascular walls. We therefore simulated a PVS segment with endfoot coverage modelled as a Voronoi tessellation with endfoot areas and circumferences matched by [37] to the *in vivo* data reported by [32]. The geometry is shown in figure 5A. The cylindrical wall of endfeet has a length of 26.5 µm and a radius of 8.5 µm, and the corresponding principal curvature is $1.2\times 10^{5}$ m${,}^{-1}$. Each endfoot is 2 µm thick and is separated from its neighbours by uniform gaps of 0.25 µm. Each endfoot is anchored at a small cylinder located near its centre on the outer surface, allowing for deformation at the edges while limiting central movement. $P_{\mathrm{PVS}}$ oscillated sinusoidally between −5 and 5 Pa with period T, which is different from the 20 Pa pressure amplitude cases presented in figures 1–3. The areas of the endfeet vary, and as a result, the distances from endfeet edges to their anchors also vary. This results in some endfeet being ‘longer’ than their neighbours. Additionally, many endfeet are ‘longer’ than the one shown in the geometry in figure 3 with larger curvature. Therefore, we chose the reduced pressure amplitude of 5 Pa to prevent numerical instability caused by excessively large deformation with complicated geometry.

Figure 5B and electronic supplementary material, video S7 show that the gaps between endfeet were widest when $P_{\mathrm{PVS}}$ was maximum (t/T=0.25). Conversely, when $P_{\mathrm{PVS}}$ was the most negative (t/T=0.75), the gaps nearly closed everywhere. Figure 5C presents the flow speed distribution at three cross-sections for t/T=0.25, showing high speed in the widened gaps. The maximum values reached 0.18 mm s^−1^.

Figure 5D shows the volume flow rate Q over one period of 0.01 Hz pressure oscillation. Fluid passed from PVS to ECS (Q>0) when $P_{\mathrm{PVS}}>0$ and from ECS to PVS (Q<0) when $P_{\mathrm{PVS}}<0$. However, the magnitude of Q was significantly greater when Q>0 than when Q<0, resulting in a net (time-averaged) flow rate $Q_{\mathrm{net}}$= $0.969\times 10^{-6}$ mm${,}^{3}$ s^−1^. Using $Q_{\mathrm{net}}$, we estimated the total CSF flow rate in the entire brain. In the human brain, assuming a penetrating arteriole density of 10 vessels mm^−^${,}^{2}$, a cortical surface area of 2000 cm${,}^{2}$, a vessel diameter of 18 µm, and a vessel length of 1 mm [38], and with a pressure amplitude of 1 Pa, the daily volume of net flow was estimated at 0.63 l.

We further examined the effect of frequency by varying it from 0.01 to 10 Hz. As shown in figure 5E, $Q_{\mathrm{net}}$ decreases slightly with increasing frequency. However, within the 0.01−10 Hz range, the decrease is small (less than 10%), indicating that flow rectification remains effective.

## 4. Discussion

Our idealized simulations suggest that wedge-shaped gaps between endfeet, pairs of long and short endfeet, and curvature of the endfoot wall can all act in a valve-like manner, favouring flow in one direction over flow in the other. In three-dimensional simulations, wedge-shaped gaps rectified flow better at corners (where three endfeet met) than at edges (where two endfeet met). Curvature rectified flow better when gaps were parallel to the axis of curvature than when they had other orientations. Future work could examine the orientations of endfoot gaps in actual tissue, to estimate the effectiveness of curvature for rectification. Of the three geometric arrangements, wedge-shaped gaps allowed the most flow and rectified best. Curvature of the wall permitted the least flow and applied the least rectification, consistent with a prediction from prior modelling that curvature produces some rectification, but not enough to match *in vivo* observations [22]. That said, since PVSs are closed spaces, curvature is essentially always present, and it could act in conjunction with wedge-shaped gaps and/or pairs of long and short endfeet. Similarly, since endfeet vary in size [39], in almost any pair of neighbouring endfeet, one is longer than the other and some corresponding rectification is possible (though the favoured direction would depend on the direction of overlap). Future work could examine the distributions of endfoot sizes in actual tissue to estimate the effectiveness of pairs of long and short endfeet for rectification. Wedge-shaped gaps require a specific shape that may be rare among real cells. Our three-dimensional simulation of a cylindrical wall of endfeet produced strong rectification without wedge-shaped gaps, thanks to the combined effects of curvature and a variation of endfoot lengths necessitated by the tiled arrangement [37].

Other valve mechanisms may also be possible. The scallop theorem dictates that viscous flow between rigid boundaries is fully reversible, making rectification impossible [40]. Though flows in PVSs are almost certainly viscous, their flexible boundaries might allow rectification, as demonstrated above. The mechanisms we have considered all respond differently to flow out of the PVS than flow into the PVS because of asymmetric shapes. Other asymmetric shapes are possible. Other sorts of asymmetry, such as endfeet whose backs are stiffer than their fronts, might also rectify flow. Such asymmetric stiffness seems to occur in lymphatic endothelial cells, which help control lymph flow and feature microtubules at concave regions and F-actin at convex lobes [31]. Beyond valves, rectification might be produced, in part or in full, by poroelasticity [41–43] and/or osmotic forces [44]. Future work could explore new rectification mechanisms and consider combinations of mechanisms.

In our simulations, wedge-shaped gaps, endfeet of unequal length, and wall curvature all rectified flow effectively over frequencies ranging from 0.01 to 10 Hz. Thus, each of the mechanisms would work equally well with fluctuations due to cardiac pulsations (approx. 5−10 Hz in mice, depending on the state of sleep, wakefulness or anaesthesia), respiration (approx. 1−4 Hz in mice), or functional hyperaemia (approx. 0.1 Hz). If putative valves had worked with only one sort of fluctuation, it would have been natural to wonder whether that sort of fluctuation was the primary driver of glymphatic flow, but according to our results, that is not the case. Rather, these valves seem to be biologically robust. In fact, their robustness to frequency variation could span species as well. Cardiac pulsations have frequencies around 1 Hz in humans and around 0.5 Hz in elephants [45]. Respiration frequencies also tend to be lower for bigger animals [46]. Functional hyperaemia frequencies are similar in rodents and humans. These valve mechanisms would work for any of those fluctuation frequencies. Glymphatic function has been observed in mice [5], rats [47], rabbits [48], pigs [49] and humans [50], so it seems to be broadly conserved across species. The valve mechanisms we have considered might be conserved as well.

One source of fluctuations at higher frequencies is ultrasound stimulation. Recent experiments showed that applying ultrasound to the brains of mice [36] and rats [35] can promote glymphatic circulation. The clinical potential is enticing; one could imagine clearing amyloid-β plaques from the brains of patients with Alzheimer’s disease using non-invasive ultrasound. That said, the mechanism is not yet understood. We wondered if the presence of valves in the glymphatic system might result in resonance frequencies, and if stimulating those frequencies might drive rapid flow. We performed two-dimensional simulations of wedge-shaped gaps, endfeet of unequal length, and wall curvature at frequencies in the ultrasound range (200–650 kHz, not shown). We found no rapid flows. In fact, these three valve mechanisms failed to rectify effectively at ultrasound frequencies. The promotion of glymphatic function by ultrasound stimulation is not explained by valves.

We have focused on fluid exiting arterial PVSs to the ECS, but the glymphatic model proposes that fluid also enters venous PVSs from the ECS. In order for wedge-shaped gaps surrounding venous PVSs to favour flow into the PVS, the gaps would have to be oriented in the opposite direction, as sketched in figure 1A, with the wide end facing the ECS and the narrow end facing the PVS. Either orientation is equally plausible, geometrically. However, to grow into one orientation around arteries and a different one around veins, endfeet would need to ‘know’ which sort of vessel they ensheath; some sort of signalling from vessel to endfoot would probably be necessary. Similarly, pairs of long and short endfeet could favour flow into venous PVSs if oriented in the opposite direction, as sketched in figure 2A. Again, to achieve different orientations around veins than arteries, signalling would seem necessary. On the other hand, curvature always favours flow out of the closed PVS, so it would hinder fluid motion from the ECS into a venous PVS. That said, because veins pulse less than arteries [51], it may be that valve action is negligible at venous PVSs, and rectification at arterial PVSs is sufficient.

The hypothesis that astrocyte endfeet function as rectifying valves implies bulk fluid flow in the ECS. Flow in the ECS cannot be measured directly with current methods, and its existence is debated. A reanalysis of *in vivo* measurements concluded that the spread of tracer dye in brain parenchyma is better modelled by a combination of diffusion and advection (tracer motion due to bulk flow) than by diffusion alone, estimating the superficial fluid velocity to be as high as 50 µm min^−1^ [52]. Modelling of *in vivo* magnetic resonance imaging of interstitial transport inferred superficial velocities around 2.7 µm min^−1^ [43]. Other studies, however, have argued that diffusion alone can explain observed tracer motion [53,54]. Though valves can act only on bulk flow, it does not need to be fast, particularly in regions far from the valves themselves. The collective ECS has a far larger cross-sectional area than the endfoot gaps (or, indeed, the entire PVSs), and mass conservation dictates that flow speed is inversely proportional to cross-sectional area, so flow is necessarily much slower in the ECS than in endfoot gaps. In much of the ECS, the bulk flow that conserves fluid mass may be too slow for tracer advection to match tracer diffusion, as has been suggested by recent modelling [55].

In our simulations, flow and valve action were driven by pressure differences across the endfoot wall with amplitudes of the order of 20 Pa. No measurements have been made of pressure differences across endfoot walls—or across any part of the glymphatic system. Brain-wide pressure differences are estimated to be of the order of 1 mmHg (133 Pa) or less [56,57], and pressure gradients in pial PVSs have been estimated to be of the order of 2 mmHg m^−1^ [58]. A 20 Pa drop across the endfoot wall, which is likely to be a high-resistance part of the system [59], seems to have the right order of magnitude. That said, the valve action described above does not depend sensitively on the pressure amplitude.

We estimated the net CSF flow in human brain to be approximately 0.64 l d^−1^ using our three-dimensional simulation of a cylindrical wall of endfeet, which produced strong rectification due to the combined effects of curvature and a variation of endfoot lengths. This value is comparable with the estimated daily CSF production of 0.5−0.6 l in healthy adults, which is primarily generated by the choroid plexuses [60]. In addition to this choroidal secretion, net fluid flow within the cortical brain tissue may arise from microvascular filtration across the blood–brain barrier and from metabolic water production. Recent modelling studies [61] suggest that total brain-wide interstitial fluid flow could range from 0.15 to 9 l d^−1^, depending on parameters such as the filtration coefficient and osmotic gradient.

To reduce simulation complexity, we used open-field boundary conditions for the fluid and did not account for the extracellular matrix. Open-field boundaries neglect downstream hydraulic impedance, which can lead to overestimated flow velocities in the gaps. More realistic resistance-compliance boundary conditions would be expected to reduce both flow velocity and endfoot deformation, and could also shift the phase of periodic flows. In addition, endfoot gaps may contain macromolecules that significantly affect fluid flow there. If a dense network of macromolecules spans the gap, viscous drag would slow fluid flowing there, and would affect fast flows more strongly than slow flows, so rectification would be reduced. On the other hand, if macromolecules tether endfeet to their neighbours and act like springs, rectification could be enhanced. Future studies that incorporate realistic resistance-compliance boundary conditions together with a characterization of the extracellular matrix in endfoot gaps and adjacent ECS will allow more precise predictions.

Many biological tissues stiffen or soften as oscillation frequency increases and are therefore well-modelled as viscoelastic, with moduli that depend on frequency. However, the microscale viscoelastic properties of endfeet have not been characterized, and the macroscale viscoelastic properties of brain tissue are debated. We considered the simple case of linear elastic endfeet, with a modulus independent of frequency. Future work could explore valve action by viscoelastic endfeet. In particular, AQP4 water channels on endfoot membranes may be an important source of viscoelasticity. In response to pressure changes, an endfoot with more AQP4 channels would inflate or deflate more quickly than one with fewer channels. Accordingly, we expect that AQP4 would make endfeet less stiff, especially at low frequencies.

The predictions of our simulations can be tested directly once imaging methods become available that can resolve endfoot gaps *in vivo*. We hope that simulated emission depletion microscopy [62] or some other method will soon make such observations possible.

## Data Availability

There are no new experimental data involved in our paper. The simulations presented in the paper were all carried out using the commercial software COMSOL, and the parameters required to reproduce these simulations are fully detailed within the manuscript. Supplementary material is available online [63].

## Appendix A. Validation and convergence

We compared our numerical results with the two-dimensional theoretical predictions in Bork et al. [3], as shown in figure 6. Good agreement was generally achieved: when $P_{PVS}$ was positive, our simulation results overlapped with analytical solution; when $P_{PVS}$ was negative, our results showed less flow. The mismatch may stem from the fact that the theoretical predictions neglected stretching and the effects of shear forces on the endfeet, which leads to early close of the gap when $P_{PVS}<0$. The threshold pressures for the valve to shut are important in flow rectification, and we found that the pressure for the valve to shut is $P_{PVS}=-0.259$ mmHg for the simulation case with large parameters and $P_{PVS}=-0.128$ mmHg for small parameters.

To verify the adequacy of spatial resolutions, mesh sensitivity studies were performed for both two- and three-dimensional simulations. All meshes were generated in COMSOL with user-defined element sizes, and second-order elements were used in all cases. Triangular elements were used in two-dimensional simulations and tetrahedral elements were used in three-dimensional simulations. Adaptive mesh refinement was not employed, as it substantially increased computational cost without improving the results. The mesh was refined near the endfoot walls, particularly in the gaps. As shown in figure 7, the flow rates Q do not change significantly in both two- and three-dimensional simulations when meshes are further refined from medium to fine. Therefore, element sizes in medium meshes were used for all simulations in this study.

To verify the adequacy of temporal resolutions, time-step convergence studies were performed. All simulations used the adaptive time stepping scheme based on the implicit backward differentiation formula. Step size was controlled by the relative tolerance, which sets the maximum allowable relative error in the solver iterations. A relative tolerance of 0.001 was used in all simulations, as further reductions produced no noticeable change in the simulation results.
